# Case Report: A Novel *PAX3* Mutation Associated With Waardenburg Syndrome Type 1

**DOI:** 10.3389/fgene.2021.609040

**Published:** 2021-03-04

**Authors:** Qiuming Hu, Huazhong Ma, Jiawei Shen, Zongming Zhuang, Jianqiang Li, Xinlan Huang, Xian Li, Haoyu Li

**Affiliations:** ^1^Department of Ophthalmology, The First Affiliated Hospital of Guangxi Medical University, Nanning, China; ^2^Department of Ophthalmology, Pingguo People's Hospital, Baise, China; ^3^Department of Stomatology, The Affiliated Hospital of Medical School, Ningbo University, Ningbo, China; ^4^Department of Retina and Vitreous, Jingliang Eye Hospital, Guangxi Medical University, Nanning, China; ^5^Division of Pharmacy and Optometry, Faculty of Biology, Medicine and Health, University of Manchester, Manchester, United Kingdom

**Keywords:** Waardenburg Syndrome Type 1, Heterochromia iridis, *PAX3* gene, whole exome sequencing, case report

## Abstract

**Background:** Waardenburg Syndrome Type 1 (WS1) is a rare hereditary disease, which is usually caused by the mutations of *PAX3* (*paired box 3*). Here, we reported a pedigree with WS1, which was caused by a novel mutation in *PAX3*.

**Case Report:** In this present report, a 10-year-old boy and his twin sister from a Han Chinese family presented with iris pigmentary abnormality, synophrys, and broad and high nasal root. Their father presented premature whitening of the hair, but no iris pigmentary abnormality. Their aunts presented the same clinical characteristics with the twins and premature graying of hair. However, none of the patients reported hearing loss. The clinical diagnosis of the four patients from this pedigree was WS1. The whole exome sequencing (WES) revealed a novel mutation (c.959-5T>G) in the *PAX3* gene, which could be responsible for the observed pathogenic of WS1 in this pedigree. The genetic test confirmed the diagnosis of WS1 in the four patients from the studied pedigree.

**Conclusion:** This present study demonstrated that genetic test based on WES, an effective alternative to regular clinical examinations, helps diagnose WS1. The newly identified *PAX3* gene mutation can expand the understanding of WS1.

## Introduction

Waardenburg Syndrome (WS), also named auditory-pigmentary syndrome, is one of the most common causes of syndromic deafness, contributing to 2–5% of congenital deafness cases. This syndrome is mainly caused by monogenetic variants that are mostly inherited through autosomal dominance with incomplete penetrance (Read and Newton, 1997). In China, WS patients account for about 1% of the deaf population (Chen et al., 2020). WS can be classified into four types (WS1, OMIM# 193500; WS2, OMIM# 193510; WS3, OMIM# 148820, and WS4, OMIM# 277580) according to different clinical characteristics, and WS1 and WS2 are the most common types (Pingault et al., 2010). The first WS case was described as a syndrome of a disorder combining anomalies of the eyelids, eyebrows, and nasal root with congenital deafness, which is now known as WS1 (Waardenburg, 1951). WS2 is similar to WS1, but hearing loss is more common in the former, and widely spaced eyes occur more common in the latter (Arias, 1971). WS3, also known as Klein-Waardenburg syndrome, often exhibits all the clinical features of WS1 and upper limb deformity. WS4, also known as Waardenburg-Hirschsprung disease, often exhibits all the clinical characteristics of WS2 in addition to megacolon or gastrointestinal atresia (Ma et al., 2019). WS is usually caused by mutations of the following six genes (Pingault et al., 2010): *PAX3* (*paired box 3*), *EDN3* (*endothelin 3*), *EDNRB* (*endothelin receptor type B*), *MITF* (*microphthalmia-associated transcription factor*), *SOX10* (*SRY Box10*), and *SNAI2* (*snail homolog 2*). *PAX3* gene mutations are the common genetic causes of WS1 and WS3 (Li et al., 2020).

WS is often described as an autosomal dominantly inherited disorder of neural crest (NC) cells. *PAX3* protein contains two highly conserved DNA binding domains (Apuzzo and Gros, 2007): a pairing domain (PD, Amino acids at position 33–160) and a homologous domain (HD, Amino acids at position 219-276). Genes, including *PAX3*, play a vital role in the development and differentiation of melanocytes derived from embryonic NC cells. The corresponding protein function will be affected, if a mutation occurs in the functional region, which will cause WS (Liu et al., 2020). Until now, over 150 mutations of the *PAX3* gene associated with WS have been reported (Li et al., 2020), and most of them are located at exons 2–6 (Pingault et al., 2010). In this report, a novel mutation (c.959-5T>G) of the *PAX3* gene was detected in a Chinese family with WS1. The discovery of this study may provide valuable information for genetic counseling of WS1 families.

## Case Presentation

**Figure 1:**
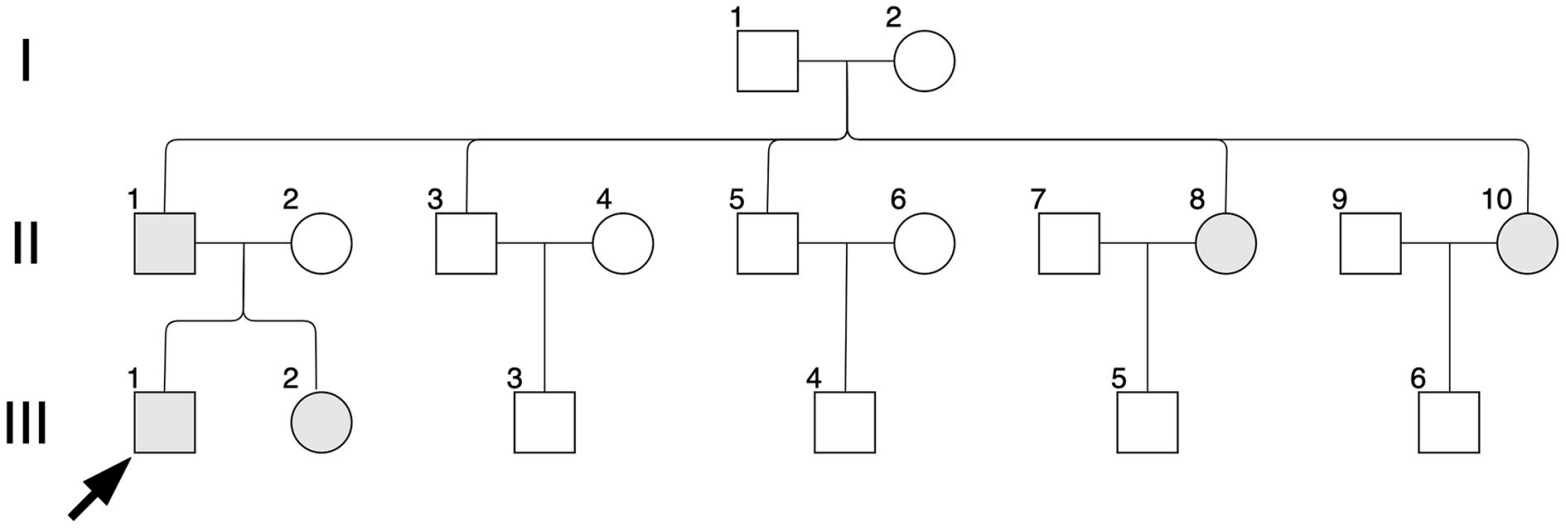
Pedigree diagram. Black arrow indicates the proband, II-1; shapes in gray indicate affected individuals, II-1, II-8, II-10, III-1 and III-2.

**Figure 2:**
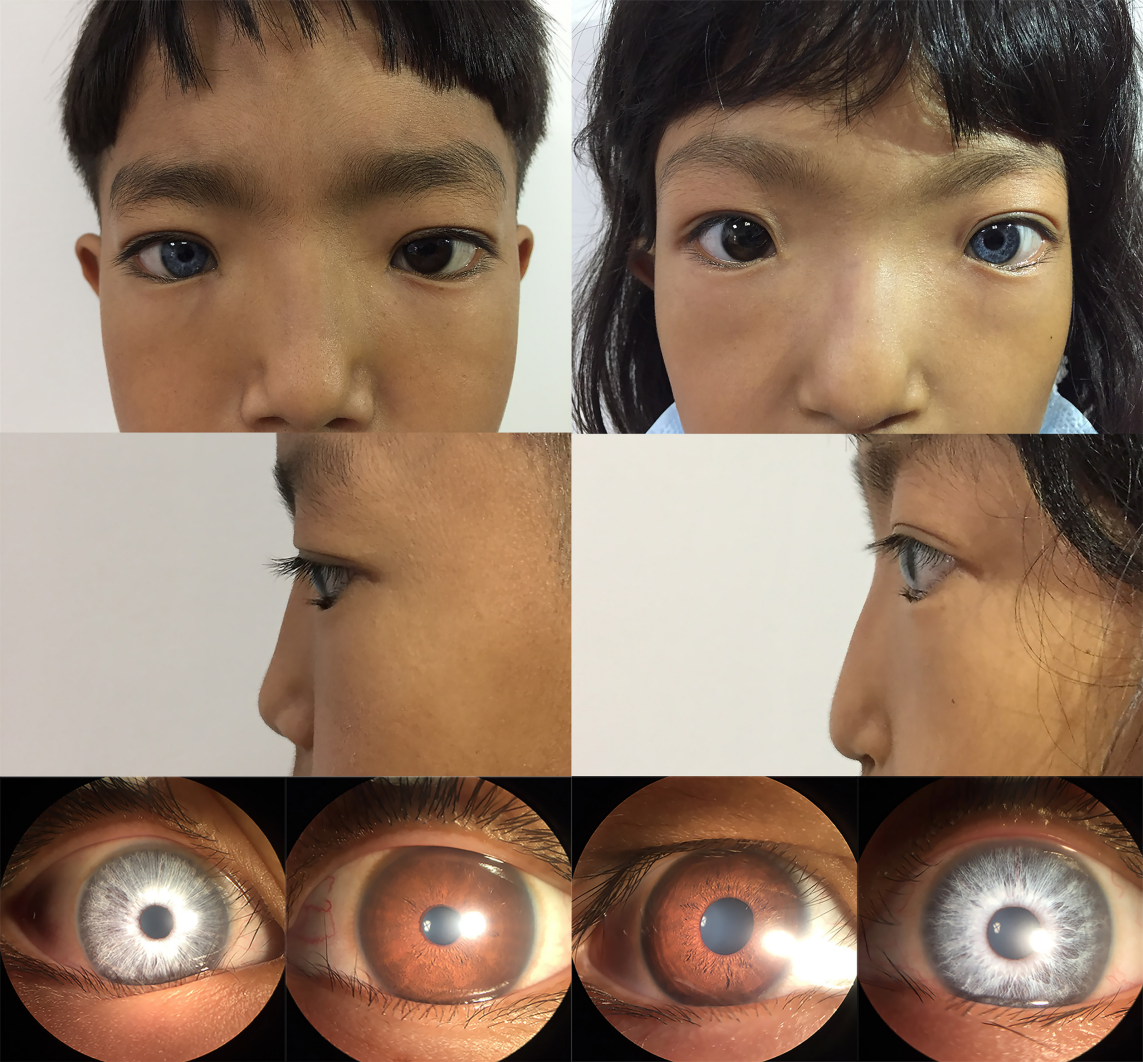
Photographic features of appearance and iris pigment in III-1 and III-2. Synophrys, and broad, and high nasal root without forehead white hair can be observed in III-1 (proband) and III-2. The iris color in the right eye of III-1 is off-white and the left eye is brown. The iris color in the right eye of III-2 is brown and the left eye is off-white.

**Figure 3:**
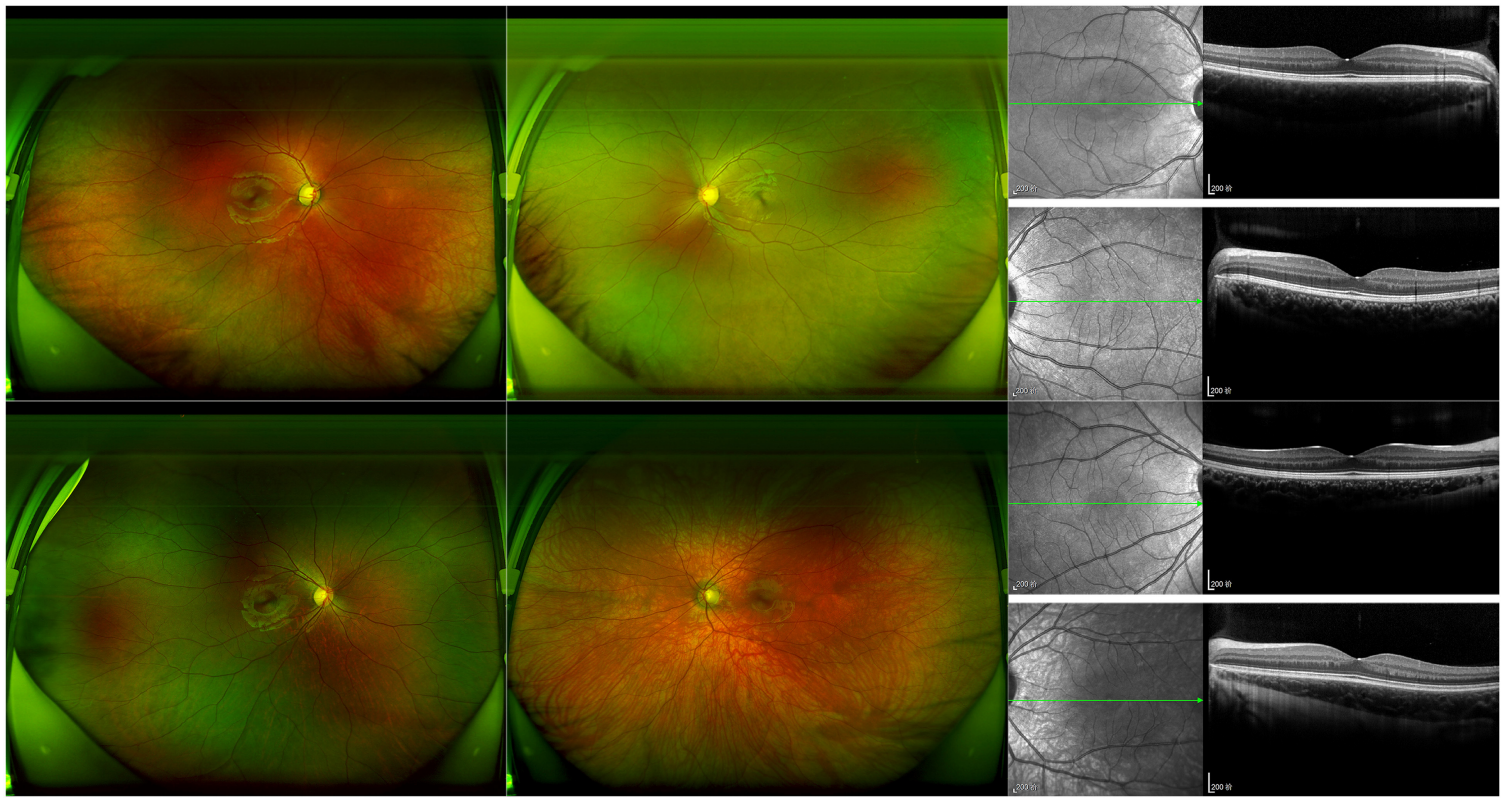
Photographic features of fundus in III-1 and III-2. Ultra-widefield laser scanning image and B-scan results of optical coherence tomography of III-1 (proband) and III-2 are shown. The hypopigmented fundus on the same side as the depigmentation of the iris can be observed.

In the studied family, a 10-year-old boy (the proband, III-1), his father (II-1), aunts (II-8 and II-10), and twin sister (III-2) presented with iris pigmentary abnormality, synophrys, and broad and high nasal root (Figure 1). This studied family members were Han Chinese and had no history of medication use, infectious disease, severe constipation, blockage of the intestine, or abnormalities or limits of mobility in the limbs. The iris pigmentary abnormality, synophrys, and broad and high nasal root of the proband were observed, the iris color in his right eye was off-white, and the color in his left eye was brown (Figure 2). Like the proband, patient III-2 was with the same clinical features, the iris color in her left eye was off-white, and the color in her right eye was brown (Figure 2). Based on iris pigmentary abnormality, synophrys, and broad and high nasal root, both II-8 and II-10 had premature whitening of hair. However, there was only premature whitening of the hair but no iris pigmentary abnormality in II-1. Notably, none of the patients in this pedigree complained of hearing loss, and they refused pure-tone audiometry. The clinical features are summarized in Supplementary Table 1. The patients III-1 and III-2 received a comprehensive ophthalmological examination, which included comprehensive medical optometry, anterior segment photography, optical coherence tomography, and ultra-widefield laser scanning imaging. Except for abnormal iris color and fundus depigmentation in the corresponding eye, no other abnormalities or refractive errors were found (Figure 3).

In addition to clinical examinations, analysis of mutations *via* whole exome sequencing (WES) using peripheral blood was performed for II-1, II-2, II-8, II-10, III-1, and III-2, which was conducted by Aegicare Technology Co., Ltd. (Shenzhen, China). The DNA was extracted from the patients' blood sample, and 20,000 exons and the region about 20 bps upstream and downstream were sequenced. The reference genome GRCh37 was used for sequence alignment. The Aegicare's Weaver algorithm was used to detect copy number variations at the exon level. The sequencing depth was over 30 × average depth. Then the sequencing results were verified through Sanger sequencing. In this study, the most major mutation associated with the clinical features located at *PAX3* (c.959-5T>G). The c.959-5T>G mutation was a novel mutation of NO. 959-5 nucleotide from thymine to guanine, which occurred in intron 6 of the *PAX3* gene. Other mutations identified in this study included *COL5A1* (c.698C>G, p.Ala233Gly), *UBE3B* (c.3086G>A, p.Arg1029His), *WDR73* (c.935G>A, p.Arg312Gln) and *ZNF469* (c.1663G>A, p.Asp555Asn and c.4910G>A, p.Arg1637Gln). The candidate variants are summarized in Table 1, and the results of Sanger sequencing are shown in Supplementary Figure 1. The identified mutation of the *PAX3* gene was found in II-1, II-8, II-10, III-1, and III-2, while II-2 (the mother of the proband) had the wild-type *PAX3*. Therefore, the *PAX3* mutation of the twins was from their father.

**Table 1:** Summary of the six candidate variants of five genes in the Waardenburg Syndrome Type 1 pedigree.

**Gene**	**PAX3 (NM_181457.4)**	**COL5A1 (NM_000093.5)**	**UBE3B (NM_130466.4)**	**WDR73 (NM_032856.4)**	**ZNF469 (NM_001367624.2)**	**ZNF469 (NM_001367624.2)**
Genomic position	chr2:223085078	chr9:137619155	chr12:109972466	chr15:85186903	chr16:88495541	chr16:88498788
Position in cDNA	c.959-5T>G	c.698C>G	c.3086G>A	c.935G>A	c.1663G>A	c.4910G>A
III-1	Heterozygous	Heterozygous	Heterozygous	Heterozygous	Heterozygous	Heterozygous
II-1	Heterozygous	Heterozygous	Heterozygous	Heterozygous	Heterozygous	Wild type
II-2	Wild type	Wild type	Wild type	Wild type	Wild type	Heterozygous
II-8	Heterozygous	NA	NA	NA	NA	NA
II-10	Heterozygous	NA	NA	NA	NA	NA
III-2	Heterozygous	Heterozygous	Wild type	Wild type	Wild type	Heterozygous

*NA, not applicable; Human genome reference: GRCh37/hg19*.

In addition to the previous publication, this *PAX3* mutation was not recorded in gnomAD_exome, gnoAD_gnome, ExAc, or 1000 Genomes Project database. A series of prediction tools were used to evaluate the possible functional impacts of mutations in this study (Ng and Henikoff, 2003; Reva et al., 2007; Chun and Fay, 2009; Schwarz et al., 2010; Shihab et al., 2013; Choi and Chan, 2015). The results of the prediction are summarized in Supplementary Table 2. According to the prediction results of SpliceAI [DS_AL (acceptor loss) score = 0.5457] (Jaganathan et al., 2019), the detected mutation has a greater possibility of affecting the splicing. A DS_AL score >2 indicates a possibility of affecting the splicing; a score >5 indicates a possibility to cause a splicing-related disease. Splice variants frequently give rise to alternative splicing and affect protein coding. Consistent with the results, the mutations identified in Ehlers-Danlos-syndrome-related gene *COL5A1* (Tuna et al., 2019; Angwin et al., 2020) and Kaufman-Oculocerebrofacial-syndrome-related gene *UBE3B* (Cheon et al., 2019; Ambrozkiewicz et al., 2020) also had a higher risk of disease. However, no results of the *PAX3* mutation were available through the prediction tools. The American College of Medical Genetics and Genomics (ACMG) guidelines were used for the interpretation of variants (Richards et al., 2015).

## Discussion

Diagnosis of WS is often established by clinical features. For WS1, the diagnosis requires two major criteria or one major plus two minor criteria (Farrer et al., 1992; Saleem, 2019). There are five major criteria: (1) congenital sensorineural hearing loss; (2) white forelock; (3) abnormal iris pigment; (4) dystopia canthorum; and (5) affected first-degree relative. The five minor criteria are (1) cutaneous hypopigmentation; (2) synophrys or medial eyebrow flare; (3) broad/high nasal root or low columella; (4) hypoplastic nasal alae; and (5) premature gray hair. In this studied family, all the patients met the criteria of WS1 diagnosis, although they presented different clinical characteristics. According to previous research, congenital sensorineural hearing loss is the most frequent clinical feature of WS1 patients (Oysu et al., 2000); however, none of the patients in this family presented this feature. In addition to hearing loss, dystopia canthorum is still considered the most reliable part for WS1, although it is a controversial diagnostic criterion (Sun et al., 2016; Minami et al., 2019). According to the Waardenburg consortium, dystopia canthorum should be evaluated by the W index. In this study, all the patients could be diagnosed with WS1 without considering this unagreeable indicator, the W index. Consistent with the previous research (Shields et al., 2013), the ocular features of III-1 and III-2 were described in this report that abnormal iris color and fundus depigmentation in the corresponding eye.

The sensorineural hearing loss is one of the typical phenotypes of WS1; however, none of the patients in this family complained of hearing loss. The rough hearing test also showed no abnormalities. Regretful, they refused the pure-tone audiometry. We deduced that the patients' hearing is generally normal, or the damage is too slight to detect.

The melanocyte, one of the cells affected by WS1, is derived from the NC. The NC can produce a series of cells and tissues, including melanocytes, neurons, the enteric nervous system, the facial skeleton, and other structures (Mica et al., 2013). Melanocytes exist in human skin, eyes, and cochlea. Melanocytes in the epidermis and iris contribute to skin and eye color variation, respectively (Lin and Fisher, 2007; Shields et al., 2013); melanocytes in the stria vascularis of the inner ear contribute to normal hearing (Tachibana, 2001). The biological activities of melanocytes are mediated by a series of genes, including *MITF, PAX3*, and other genes (Tachibana et al., 1996). MITF protein is essential for the survival and function of melanocytes, while transcription factors, such as *PAX3*, regulate *MITF* expression *via* extracellular signaling (Hou and Pavan, 2008). Additionally, *PAX3* is broadly expressed in several other lineages of NC cells, and early expression of *PAX3* is critical for developing melanocytes, craniofacial structure, and formation of the upper limbs (Wildhardt et al., 2013). Therefore, it is not difficult to understand that the abnormal expression of *MITF* or its regulatory genes can lead to WS characterized by depigmentation. In this study, the *PAX3* gene mutation (c.959-5T>G) was identified through WES and Sanger sequencing, and it was considered the cause of WS1.

In this study, several mutations were discovered. According to ACMG guidelines and clinical manifestations, mutations with lower risk or inconsistent characteristics were excluded. Consistent with the clinical phenotypes and genetic traits, the proband's *PAX3* conversion came from his father and was inherited autosomal dominantly. However, this novel mutation of *PAX3* was rare, and due to the low frequency of population database and the mutation was co-segregated with the disease in multiple family members, according to the ACMG guidelines, it was classified as a variant of uncertain significance (VUS). Further study is needed to verify our results.

Comprehensive management of patients is required. As WS1 is a high-risk indicator for hearing loss, hearing screening and auditory diagnostic assessments are needed. In addition to the auditory system, wearing contact lenses or sunglasses to relieve photophobia and appropriate integumentary system protection and treatments are necessary. A previous study indicated that the pathogenic *PAX3* alleles might increase the risk of severe neural tube defects in the patients' offspring, associated with folate-response (Hart and Miriyala, 2017). Thus, daily folic acid supplementation is recommended to all childbearing age women (Saleem, 2019).

In conclusion, this present study reports a novel mutation, c.959-5T>G of the *PAX3* gene in a Han Chinese family with WS1. Discovering and reporting novel WS1-associated mutations facilitate the analysis of correlations between WS genotypes and phenotypes, helping further genetic consultation and diagnosis. Besides, our results showed that WES is a useful approach for congenital disease diagnosis and is of great benefit to disease screening, genetic diagnosis, and counseling.

## Data Availability Statement

The datasets for this report are not publicly available due to concerns regarding participant anonymity. The datasets generated for this study can be available on request to the corresponding author.

## Ethics Statement

The studies involving human participants were reviewed and approved by The Ethics Committee of Jingliang Eye Hospital Affiliated to Guangxi Medical University. Written informed consent was obtained from the minor(s)' legal guardian/next of kin for the publication of any potentially identifiable images or data included in this article.

## Author Contributions

HM, JL, XH, and ZZ cared for the patients and performed medical examinations. QH took the lead in writing the manuscript. HM, JS, XL, and HL revised the manuscript. XL and HL designed the present research and guided the entire essay. All authors reviewed the manuscript and provided critical feedback and agreed on the final manuscript.

## Conflict of Interest

The authors declare that the research was conducted in the absence of any commercial or financial relationships that could be construed as a potential conflict of interest.

## Supplementary Material

Supplementary Figure 1 The c.959-5T>G mutation in the PAX3 gene, which is identified in III-1 (proband), II-1, II-8, II-10 and III-2. The wild type is identified in II-2.
