# Supplementary material for: Case Report: A Novel PAX3 Mutation Associated With Waardenburg Syndrome Type 1
Source: Front Genet. 2021 Mar 4;12:609040. doi: 10.3389/fgene.2021.609040 (PMC7970110; doi:10.3389/fgene.2021.609040)
Supplement: Supplementary file 1 [file Data_Sheet_1.docx]

TABLE S1 Clinical characteristics associated with Waardenburg Syndrome Type 1 of patients in pedigree.

| Patient ID | II 1 | II 8 | II 10 | III 1 | III 2 |
| --- | --- | --- | --- | --- | --- |
| Sex | Male | Female | Female | Male | Female |
| Hearing loss | - | - | - | - | - |
| Iris pigmentary abnormality | - | + | + | + | + |
| Forehead white hair | - | - | - | - | - |
| Abnormal skin pigmentation | - | - | - | - | - |
| Synophrys | + | + | + | + | + |
| Broad and high nasal root | + | + | + | + | + |
| Premature greying of hair | + | + | + | - | - |
| Clinical diagnosis | WS1 | WS1 | WS1 | WS1 | WS1 |
| Genetic Diagnosis | WS1 | WS1 | WS1 | WS1 | WS1 |

TABLE S2 Predicted effect of the mutations on protein structure by multiple algorithms.

| Gene | Genomic position | Position in cDNA | SIFT | ClinPred | LRT | MutationTaster | MutationAssessor | FATHMM | PROVEAN |
| --- | --- | --- | --- | --- | --- | --- | --- | --- | --- |
| PAX3  (NM_181457.4) | chr2:223085078 | c.959-5T>G | NA | NA | NA | NA | NA | NA | NA |
| COL5A1  (NM_000093.5) | chr9:137619155 | c.698C>G | Deleterious | Deleterious | Unknown | Disease-causing | Medium | Tolerated | Deleterious |
| UBE3B  (NM_130466.4) | chr12:109972466 | c.3086G>A | Deleterious | Deleterious | Deleterious | Disease-causing | Medium | Tolerated | Deleterious |
| WDR73  (NM_032856.4) | chr15:85186903 | c.935G>A | Tolerated | Tolerated | NA | Polymorphism | Neutral | Tolerated | Neutral |
| ZNF469  (NM_001367624.2) | chr16:88495541 | c.1663G>A | Deleterious | Tolerated | NA | Polymorphism | Medium | Tolerated | Neutral |
| ZNF469  (NM_001367624.2) | chr16:88498788 | c.4910G>A | Tolerated | Tolerated | NA | Polymorphism | Neutral | Tolerated | Neutral |

NA: not applicable.
