# Supplementary figures and images for: Case Report: A Novel PAX3 Mutation Associated With Waardenburg Syndrome Type 1
Source: Front Genet. 2021 Mar 4;12:609040. doi: 10.3389/fgene.2021.609040 (PMC7970110; doi:10.3389/fgene.2021.609040)

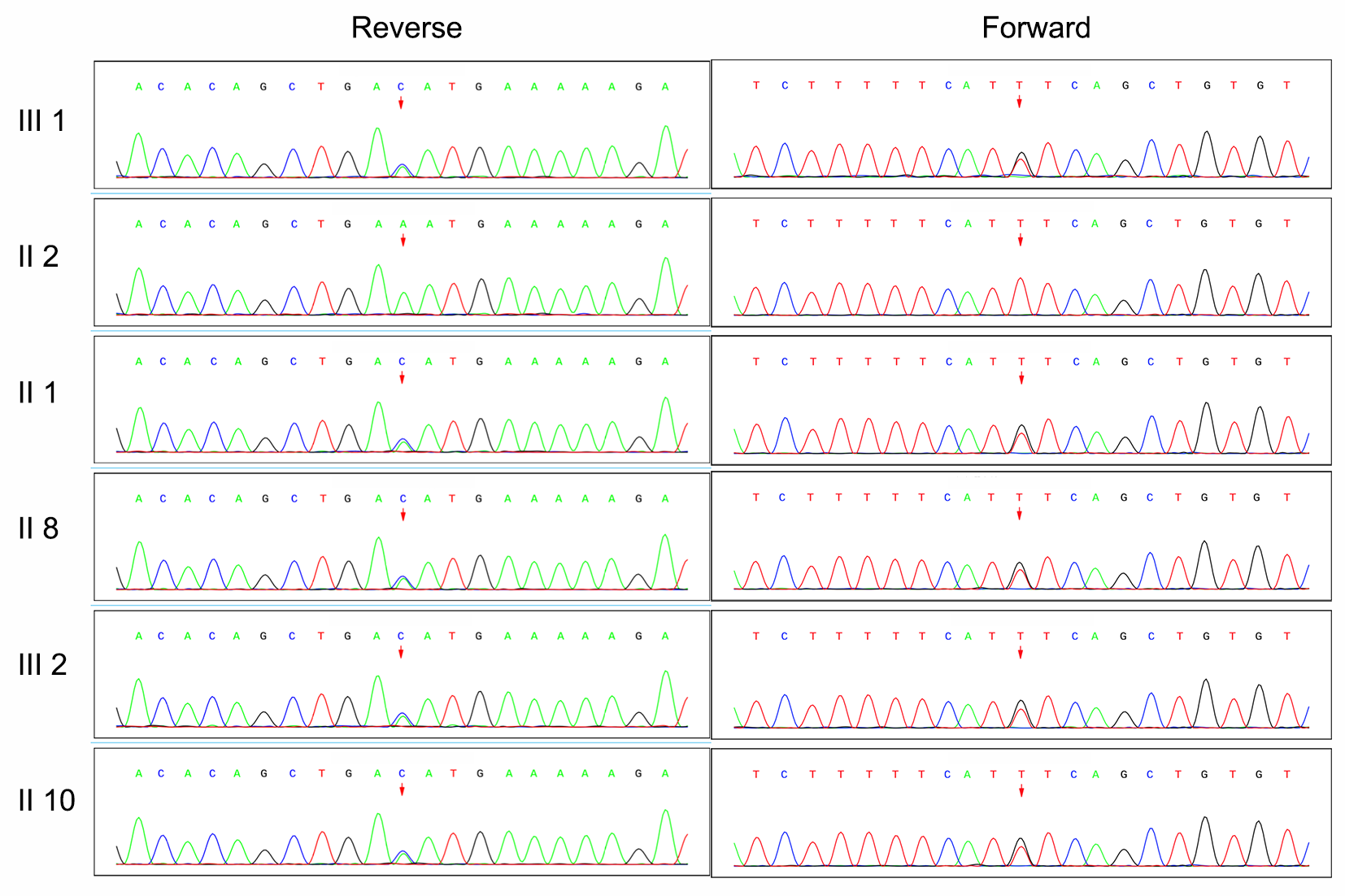

Supplement: Supplementary Figure 1 — The c.959-5T>G mutation in the PAX3 gene, which is identified in III-1 (proband), II-1, II-8, II-10 and III-2. The wild type is identified in II-2. [file Image_1.tif]
